# Oral Healthcare Utilization Factors Shaping the Perceived Oral Health Outcome Among Gond Tribes of Chhattisgarh: A Cross-Sectional Study Based on Andersen's Behavioral Model

**DOI:** 10.7759/cureus.55957

**Published:** 2024-03-11

**Authors:** Niharika Benjamin, Vishakha Rani, Bedkekar Sushma, Rohini Sharma, Aditya Purushottam Burile, Elashri Chatterjee

**Affiliations:** 1 Department of Public Health Dentistry, Hitkarini Dental College and Hospital, Jabalpur, IND; 2 Department of Public Health Dentistry, Dr. B.R. Ambedkar Institute of Dental Sciences, Patna, IND; 3 Department of Public Health Dentistry, M.R. Ambedkar Dental College and Hospital, Bangalore, IND; 4 Department of Medical Affairs, Sekhmet Technologies Private Limited, Gurugram, IND; 5 Department of Public Health, Lok Biradari Prakalp Hospital, Gadchiroli, IND; 6 Department of Periodontology, Hitkarini Dental College and Hospital, Jabalpur, IND

**Keywords:** predisposing health belief, oral health quality of life, healthcare utilization, gond tribes, dental caries, andersen’s behavioral model

## Abstract

Introduction: The Gonds are a highly ancient and expansive tribal community, ranking among the largest in the world. A review of the literature has suggested that they are more vulnerable to oral diseases and are less inclined to utilize oral health services due to the comprehensive approach that considers the socioeconomic, cultural, and structural factors affecting the Gond community's access to oral health services. Tribal health requires action in the health sector. Utilization is an essential marker of the health status of any population and is necessary to bridge the gap between tribes and the wider portion of the community. Hence, this study was conducted among the Gond tribes of Chhattisgarh to evaluate the oral healthcare utilization factors shaping the perceived oral health outcome using Andersen's behavior model.

Materials and methods: This cross-sectional study was carried out among 400 Gond tribes residing in villages of Chhattisgarh. Data was collected through a standardized questionnaire, adapted from Andersen's behavioral model of healthcare utilization during house-to-house survey. The questionnaire included predisposing, enabling, perceived, and evaluated need factors. Oral health status for evaluated need was assessed using the World Health Organization (WHO) Oral Health Assessment form (1997), and the perceived oral health outcome was measured using Oral Health Impact Profile-14 (OHIP-14). Results were computed using descriptive statistics, chi-square test, and one-way analysis of variance (ANOVA). Multivariate analysis was done using binomial logistic regression.

Results: The dental visit in the past one year was only 14%. The findings of logistic regression revealed that the perceived oral health outcome was significantly associated with age, occupation, and positive belief in the efficacy of dentist, perceived need, and presence of dental caries.

Conclusion: The findings of the present study support Andersen's behavioral model and suggest that there is an interrelationship of predisposing characters, predisposing health beliefs, and enabling need factors that determine the likelihood of use of services, which in turn determines the good or bad oral health outcome.

## Introduction

There has been a notable enhancement in healthcare facilities over the years. Amid the significant advancements made globally, there remain those who reside in seclusion, preserving their ancestral principles, practices, beliefs, and myths, and are commonly referred to as tribes. Worldwide, there are 350-370 million indigenous people in over 90 countries [1]. In India, there are 705 tribal groups, accounting for around 8.61% of the nation's total population. These groups consist of 104.28 million people and inhabit almost 15% of the land area of the nation. Chhattisgarh is the homeland of nearly 33 tribal groups constituting 31.8% of the total population [2]. Manendragarh-Chirmiri-Bharatpur (MCB) is one of the districts in Chattisgarh that has 131 villages and major tribes inhabit these villages. The original inhabitants of the Manendragarh (MCB) district were the Kols, Gonds, and Bhuinhars [3].

The Gonds are a highly ancient and exceptionally vast tribal community globally. The medical care of disease has been closely linked to the shared beliefs, conventions, traditions, values, and behaviors related to health and disease. Within the Gond community, a multitude of folktales exist, some of which pertain to matters of health. The accumulation of medical knowledge and healthcare procedures among tribal communities throughout history is referred to as 'traditional healthcare systems' or 'indigenous health practices'. These systems encompass both herbal remedies and psychosomatic approaches to therapy. This activity has consistently incorporated elements of mysticism, the paranormal, and magic, frequently culminating in distinct magico-religious rituals. These convictions should not be disregarded as ordinary superstitions but rather require thorough review due to their significant impact on diseases, managing illnesses, and the utilization of healthcare facilities [4].

Studies related to the oral health status of tribes have reported dominance of untreated caries, high prevalence of calculus, dental fluorosis, periodontal disease [5], high prevalence of tobacco use, and associated high prevalence of leukoplakia that demands high treatment needs [5-7]. The tribes are more vulnerable to diseases and are less likely to utilize health services due to socioeconomic, cultural, and structural factors affecting the Gond community's access to oral health services. This is exacerbated by the limited knowledge of the necessary precautions to safeguard their well-being, individual beliefs and customs, their geographical remoteness from medical centers, the absence of dependable transport routes, and financial limitations [5,7]. According to Mooney, access is primarily determined by the availability of resources, whereas utilization is influenced by both the availability of resources and the level of demand. The utilization of care among people can be influenced by many different variables that are unrelated to healthcare itself but influence a person's desire for health services [8]. Andersen's model is widely recognized as one of the most prominent frameworks for studying healthcare usage [9]. Throughout the years, this model went through numerous adjustments and was selected considering its ability to determine the most prognostic characteristics for dental utilization. These elements may be employed to guide targeted efforts to improve care, and multiple variables may be modified and intervened upon [10].

The model claims that certain individuals exhibit a higher propensity to utilize medical services, which can be attributed to specific settings: (i) Predisposing components, which generally consist of individual demographic traits; (ii) enabling variables that pertain to the resources accessible to an individual for acquiring services, such as income, dental insurance, and having a designated dental provider; and (iii) the need component, which encompasses both the subjective perception and the evaluation of health status by medical professionals [11]. The correlation among these three kinds of contextual elements ultimately decides the probability of service utilization. In the late 1960s, Andersen proposed in the updated model that health outcomes and satisfaction with care can be influenced by health practice and service utilization. The approach offers a scientific comprehension of many health outcomes and the factors that influence them, including the social, cultural, behavioral, and attitudinal determinants [9].

Utilization is a crucial measure of the well-being of many people. This information is required to connect the culturally distinct and socially separated Gond tribes with the rest of the society. Understanding this is needed for the overall improvement of their health status and oral health in particular. Moreover, there is no information available regarding oral healthcare utilization among the tribal population. Hence, this study was conducted among Gond tribes of Chhattisgarh to assess the oral healthcare utilization factors shaping the perceived oral health outcome using Andersen's behavioral model.

## Materials and methods

The study population comprised of Gond tribes of Chhattisgarh residing in the villages of Manendragarh (MCB) district and was carried out from April 2023 to June 2023. The study was approved by the Institutional Ethical Committee, M.R. Ambedkar Dental College and Hospital (approval no. MRADC&H/ECIRB/0827/2016-17), and written informed consent was obtained from the participants.

Eligibility criteria

Individuals who were native or permanent residents of Manendragarh (MCB) district of Chhattisgarh between the ages 15 to 55 years and above, living in the area for more than 10 years, and could comprehend the questionnaire were included. The migrants of other tribes and chronically ill patients with limited movement who were absent on the examination day and not willing to cooperate or give consent to the oral examination were excluded.

Data collection

Data was gathered through in-person interviews utilizing a questionnaire, which was subsequently followed by a clinical examination.

The sample size for the study involving the Gond tribes of Chhattisgarh residing in the villages of Manendragarh (MCB) district between April 2023 and June 2023 was determined using the desired confidence level of 95% and an acceptable margin of error of 5%. The required sample size was calculated based on the estimated prevalence rate of dental issues in the Gond population at about 30%. The calculated sample size was approximately 305 individuals. Adjustments were likely made to account for potential non-response or exclusion criteria, ensuring the final sample size sufficiently represented the target population and provided statistically reliable results.

Questionnaire

A structured closed-ended questionnaire (proforma) in the tribal local language was developed to record the data that consisted of seven sections based on the type of questions in each section. Section I consisted of socio-demographic information, section II consisted of information on predisposing health beliefs, section III consisted of information regarding the enabling resources, section IV consisted of information regarding the perceived need for oral health, section V consisted of the usage of dental services, section VI included perceived oral health outcome, and section VII included evaluated need through measurement of oral health status.

Section I: Predisposing factors of utilization: The predictors of utilization were measured by taking into account socio-demographic variables such as age, gender, education, marital status, occupation, and socioeconomic background. The study included individuals who were 15 years of age or older. These participants were categorized into five age groups: 15-24, 25-34, 35-44, 45-54, and 55 years and older. Educational attainment was evaluated based on the greatest possible degree of qualification obtained, which includes a degree or higher, education under the degree level, or no formal education. The occupation was evaluated as either unemployed, lacking specialized skills, possessing a certain degree of skill, owning a shop or being a farmer, or having a semi-professional or professional career. Following the recording of the family's monthly earnings as per capita income and the overall number of household individuals, the socioeconomic class was determined using a modified Prasad et al. classification of socioeconomic class (2017). The study condensed the groups into three: upper class, middle class, and lower class [12].

Section II: Predisposing health belief: The questionnaire consisted of eighteen items assessing oral health beliefs. Each item encompassed five groupings of responses. Reverse coding was applied to all the questions with negative wording. According to previous research conducted by Chen and Tatsuoka, the 18 items related to oral health beliefs were categorized into six factors. These factors include the perceived seriousness of disease (rated on a scale of 4-20), the perceived importance of oral health (rated on a scale of 3-15), the betterment of preventive practices (rated on a scale of 2-10), the efficacy of dentists (rated on a scale of 2-4), barriers (rated on a scale of 6-35), and motivation [13].

Oral health beliefs encompassing attitudes, perceptions, and understanding of oral health significantly influence individuals' willingness to seek dental care, adhere to treatment plans, and engage in preventive behaviors. Positive oral health beliefs often correlate with trust in the competence and effectiveness of dentists, leading to regular dental visits and better treatment compliance. Conversely, negative oral health beliefs may result in hesitancy to seek care, treatment delays, or dissatisfaction with dental services.

Section III: Enabling factors of utilization of dental services: Enabling factors were measured as oral health education advice, awareness about dentists, availability of dental service, transport facilities, perceived treatment expense, and dental anxiety.

Section IV: Perceived need for oral health: The perceived need was assessed by a three-point scale (Good; Fair; Poor).

Section V: Use of dental services: The inquiries "Have you visited a dentist within the past 12 months?" were used to evaluate the frequency of past dental visits and the individual's familiarity with dental appointments (Yes; No). The purpose of dental visits was evaluated based on the question "In general, why do you seek dental care?" (a routine appointment; an occasional appointment; solely when experiencing dental issues).

Section VI: Perceived oral health outcome: The Oral Health Impact Profile-14 (OHIP-14) evaluates the occurrence of issues related to the oral cavity or dentures across seven parameters: functional constraints, pain, psychological discomfort, physical impairment, psychological impairment, social impairment, and handicap. Participants are requested to evaluate each item during the past three months using a five-point scale. The combined effect of responses to items 1-2, 3-5, and 7-8 represented the physical function; the sum of items 5-6 and 9-10 represented the psychological function; and the sum of items 11-12 and 13-14 represented the social function.

Section VII: Objective need: The objective need was assessed by the professional using the World Health Organization (WHO) proforma 1997. The variables such as community periodontal index (CPI) Index, attachment loss, dentition status, prosthetic status, and treatment needs parameters were included.

Clinical Examination

The clinical examination was done at the participant’s home, and it was carried out under natural or artificial lighting.

Training and calibration of the investigator: A single researcher performed the participant examinations. The process of intra-examiner standardization involved the evaluation of 400 participants proceeded by their revisiting one week later. This contributed to a diagnostic acceptance rate of 85% and the kappa statistic of 0.82. The investigator was trained to record WHO 1997 oral health status proforma before the commencement of the study. The instruments were autoclaved in a private dental clinic in that particular area and carried in a sterile pouch to the site of examination. It took about five minutes for the clinical examination and about 20 minutes for the questionnaire to be answered by the participants. The assistant was trained to help the investigator while interviewing the participants.

Statistical analysis

The statistical analysis was performed using Statistical Package for the Social Sciences (IBM SPSS Statistics for Windows, IBM Corp., Version 25, Armonk, USA). The data was computed employing the Mean ± SD, and the quantitative factors were analyzed statistically using an analysis of variance (ANOVA). The categorical factors were evaluated using the chi-square test. Binary logistic regression analysis was applied to appraise the association between age, education, socioeconomic status, enabling factors, the perceived need for oral health, decayed, missing, and filled teeth (DMFT), prosthetic need in the maxillary arch, past dental visits, and perceived oral health outcomes. A P-value less than or equal to 0.05 was considered statistically significant.

## Results

The study was conducted on 400 Gond tribes inhabiting the confined villages of Chhattisgarh. The sample consisted of 47.8% (n=191) males and 52.3% (n=209) females, belonging to 15-55 years of age with a mean age of 34.84 ± 12.2. Predisposing factors consisted of age, gender, education, occupation, socioeconomic status, and predisposing health beliefs. Most participants, a total of 182, had not attended school (45.5%), 309 were married (77.3%), and 131 were farmers or shop owners (32.8%). Few, around 26, had completed higher education (6.5%). 

There was no significant association found between the age group and reason to visit (P=0.168) with a majority (99%) of subjects reporting that they visit the dentist only when there is a problem and belonged to the 15-24 years age group, and only 1.4% of subjects who belonged to 25-34 years of age group reported that they visit a dentist for regular checkup. There was no significant association found between gender and reason to visit the dentist (P=0.209). Females (97.6%, n=204) reported the reason to visit the dentist as only when there is a dental problem, and 95.8% (n=183) of males reported at checkups when there was a problem. However, significant differences were noted between reason to visit and education, occupation, and socioeconomic status (P<0.05) (Table 1).

**Table 1:** Association between predisposing socio-demographic characters and reason to visit the dentist n: Number of participants

Age group	Reason to visit the dentist						P-value
	Regular visit		Occasional visit		When in problem		
	n	%	n	%	n	%	
15-24	0	0.0	1	1.0	95	99.0	0.168
25-34	2	1.4	2	1.4	141	97.2	
35-44	0	0.0	4	4.2	91	95.8	
45-54	0	0.0	2	6.7	28	93.3	
55 and above	0	0.0	2	5.9	32	94.1	
Gender							
Male	2	1.0	6	3.1	183	95.8	0.209
Female	0	0.0	5	2.4	204	97.6	
Education							
No schooling	0	0.0	6	3.3	176	96.7	0.019
Schooling below degree level	0	0.0	4	2.1	188	97.9	
Degree or above	2	7.7	1	3.8	23	88.5	
Marital status							
Married	1	0.3	10	3.2	298	96.4	0.343
Unmarried	1	1.1	1	1.1	89	97.8	
Occupation							
Unemployed	0	0.0	5	3.5	137	96.5	0.036
Semi-skilled worker	0	0.0	0	0.0	73	100	
Skilled worker	0	0.0	1	3.2	31	96.8	
Shop owner and farmer	0	0.0	4	3.0	127	97	
Professional	2	9.1	1	4.5	19	86.4	
Socioeconomic status							
Upper class	1	1.3	5	6.3	74	92.5	0.013
Middle class	1	0.4	2	0.8	260	98.9	
Lower class	0	0.0	4	7.0	53	93.0	

No significant association was found between periodontal disease (P=0.842) or loss of attachment (P=0.860) and dental visits in the past year. However, a highly significant association was observed between dental caries and dental visits (P=0.006), with 17.8% (n=43) of caries patients visiting the dentist compared to 91.8% (n=146) of those without caries. The prosthetic status of the maxillary arch showed no significant association with dental visits (P=0.524), whereas the mandibular arch exhibited a highly significant association (P=0.001), with all prosthetic patients visiting the dentist. Additionally, a significant association was found between prosthetic need in both arches and dental visits (P<0.01), with 25% (n=18) and 25.8% (n=23) of patients needing prosthetics in the maxillary and mandibular arches, respectively, upon visiting the dentist in the past year (Table 2).

**Table 2:** Association between oral health status and dental visits in the past one year n: Number of participants; CPI: Community periodontal index status; DMFT: Decayed, missing, filled teeth index

Variables		Past dental visits				P-value
		Yes		No		
		n	%	n	%	
CPI status	Absent	9	13.2	59	86.8	0.842
	Present	47	14.2	285	85.8	
Loss of attachment	Absent	23	14.4	137	85.6	0.860
	Present	33	13.8	207	86.3	
DMFT	Absent	13	8.2	146	91.8	0.006
	Present	43	17.8	198	82.2	
Prosthetic status in the maxillary arch	Absent	55	13.9	341	86.1	0.524
	Present	1	25.0	3	75.0	
Prosthetic status in the mandibular arch	Absent	54	13.6	344	86.4	0.001
	Present	2	100	0	0.0	
Prosthetic need in the maxillary arch	Absent	38	11.6	290	88.4	0.003
	Present	18	25	54	75	
Prosthetic need in the mandibular arch	Absent	33	10.6	278	89.4	0.001
	Present	23	25.8	66	74.2	

There was a high statistically significant association found between the barrier (P=0.001), total predisposing health belief (P=0.021), and reason to visit the dentist. There was no significant association found between the perceived seriousness of disease (P=0.144), perceived importance of oral health (P=0.058), benefits of preventive practices (P=0.075), efficacy of dentist (P=0.829), and motivation (P=0.269) (Table 3).

**Table 3:** Association between predisposing health belief and reason to visit the dentist SD: Standard deviation

Predisposing health belief	Reason to visit the dentist			P-value
	Regular visit	Occasional visit	When in problem	
	Mean±SD	Mean±SD	Mean±SD	
Perceived seriousness of disease	4.00±0.00	3.45±0.47	3.63±0.39	0.144
Perceived importance of oral health	4.00±0.00	3.48±0.40	3.68±0.33	0.058
Benefits of preventive practices	3.00±1.41	3.63±0.50	3.67±0.40	0.075
Efficacy of dentist	4.00±0.00	3.81±0.40	3.83±0.39	0.829
Barriers	4.00±0.00	2.9±0.40	3.00±0.37	0.001
Motivation	4.00±0.00	3.81±0.60	3.55±0.65	0.269
Total predisposing health beliefs	3.88±0.15	3.36±0.28	3.45±0.24	0.021

There was a statistically significant association found between the age group and perceived oral health outcome (P=0.022), with a maximum of 96.7% of subjects in the age group of 45-54 years perceived good oral health outcome. The majority (17.9%, n=17) of subjects in the 35-44 years age group reported poor perceived oral health outcomes (Table 4).

**Table 4:** Association between predisposing socio-demographic characters and perceived oral health outcome n: Number of participants

Age group	Perceived oral health outcome				P-value
	Good		Poor		
	n	%	n	%	
15-24	86	89.6	10	10.4	0.022
25-34	135	93.8	9	6.3	
35-44	78	82.1	17	17.9	
45-54	9	96.7	1	3.3	
55 and above	28	82.4	6	17.6	
Gender					
Male	175	92.1	15	7.9	0.077
Female	181	86.6	28	13.4	
Education					
No schooling	167	91.8	15	8.2	0.038
Schooling degree level	169	88.5	22	11.5	
Degree or above	20	76.9	6	23.1	
Marital status					
Married	270	87.7	38	12.3	0.065
Unmarried	86	94.5	5	5.5	
Occupation					
Unemployed	131	92.3	11	7.7	0.001
Semi-skilled worker	59	80.8	14	19.2	
Skilled worker	29	90.6	3	9.4	
Shop owner and farmer	122	93.8	8	6.2	
Professional	15	68.2	7	31.8	
Socioeconomic status					
Upper class	70	87.5	10	12.5	0.132
Middle class	239	91.2	8.8	10	
Lower class	47	82.5	10	10.8	

Among the domains of predisposing health belief, only the efficacy of the dentist was found to have a significant association (P=0.018) with perceived oral health outcomes. Whereas there was no significant association found between the perceived seriousness of disease (P=0.927), perceived importance of oral health (P=0.799), benefits of preventive practices (P=0.813), barriers (P=0.918), motivation (P=0.842), and perceived oral health outcome (Table 5).

**Table 5:** Association between predisposing health belief and perceived oral health outcome SD: Standard deviation

Predisposing health belief	Perceived oral health outcome		P-value
	Good	Bad	
	Mean±SD	Mean±SD	
Perceived seriousness of disease	3.98±0.38	3.63±0.40	0.927
Perceived importance of oral health	3.16± 1.02	3.12 ±0.89	0.799
Benefits of preventive practices	3.67±0.42	3.66±0.41	0.813
Efficacy of dentist	3.83±0.37	3.69 ±0.51	0.018
Barriers	3.01±0.37	3.02±0.38	0.918
Motivation	3.54±0.64	3.69±0.74	0.151
Total predisposing health beliefs	3.45±0.25	3.45±0.23	0.842

There was a statistically significant association found between the enabling factor of oral health utilization and perceived oral health outcome (P=0.036), with a maximum, i.e., 91.2% (n=208) of participants who found that there are no resources and perceived good oral health outcome and the majority, i.e., 14.2% (n=19) of participants reported poor resources and perceived poor oral health outcome (Table 6).

**Table 6:** Association between enabling factors and perceived oral health outcome n: Number of participants

Enabling resources	Perceived oral health outcome				P-value
	Good		Poor		
	n	%	n	%	
No resources	208	91.2	20	8.8	0.036
Poor resources	115	85.5	19	14.2	
Fair resources	33	89.2	4	10.8	
Total	356	89.2	43	10.8	

There was a high statistically significant association found between the perceived need for oral health and perceived oral health outcome (P=0.000), with a maximum, i.e., 94.0% (n=79) of subjects who reported good perceived oral health outcome also found their oral condition as good and the majority, i.e., 27.4% (n=17) of subjects who reported poor perceived oral health outcome also found their oral condition as poor (Table 7).

**Table 7:** Association between perceived need for oral health and perceived oral health outcome n: Number of participants

Perceived need for dental treatment	Perceived oral health outcome				P-value
	Good		Poor		
	n	%	n	%	
Good	79	94.0	5	6.0	0.001
Fair	232	91.7	21	8.3	
Poor	45	72.6	17	27.4	
Total	356	89.2	43	10.8	

There was no significant association between the loss of attachment and perceived oral health outcome (P=0.345), with a maximum, i.e., 90.4% (n=217) of subjects who had a loss of attachment reported good perceived oral health outcome and a maximum of 12.6% of subjects who did not have a loss of attachment reported poor perceived oral health outcome (Table 8).

**Table 8:** Association between oral health status and perceived oral health outcome n: Number of participants; CPI: Community periodontal index status; DMFT: Decayed, missing, and filled teeth index

Oral health status		Perceived oral health outcome				P-value
		Good		Poor		
		n	%	n	%	
CPI status	Absent	60	88.2	8	11.8	0.773
	Present	296	89.4	35	10.6	
Loss of attachment	Absent	139	87.4	20	12.6	0.345
	Present	217	90.4	23	9.6	
DMFT	Absent	149	93.7	10	6.3	0.019
	Present	207	86.3	33	13.8	
Prosthetic status in the maxillary arch	Absent	353	89.4	42	10.6	0.357
	Present	3	75.0	1	25.0	
Prosthetic status in the mandibular arch	Absent	355	89.4	42	10.6	0.073
	Present	1	50	1	50	
Prosthetic need in the maxillary arch	Absent	340	89.9	38	10.1	0.048
	Present	16	76.2	5	23.8	
Prosthetic need in the mandibular arch	Absent	341	89.7	39	10.3	0.139
	Present	15	78.9	4	21.1	

The findings of the multivariate analysis indicated that irrespective of the use of dental services many predisposing socio-demographic characteristics, predisposing health beliefs, and perceived and evaluated needs had their effect on perceived oral health outcomes. Their overall explanatory power was R^2^ = 0.365, i.e., 36.5% of the deviance in dental visits in the past one year could explain these factors (Table 9).

**Table 9:** Binary logistic regression analysis showing the association between age, education, socioeconomic status, enabling factors, perceived need for oral health, DMFT, prosthetic need in the maxillary arch, past dental visits, and perceived oral health outcome DMFT: Decayed, missing, and filled teeth index

Variables	P-value	Odds ratio	95% Confidence interval		
			Upper bound	Lower bound	
Age (reference 55 and above)	0.022				
15-24 years	0.993	0.99	0.16		5.89
25-34 years	0.218	0.35	0.06		1.84
35-44 years	0.689	1.37	0.28		6.55
45-54 years	0.049	2.8	0.08		0.99
Education (reference degree level or above)	0.319				
No schooling	0.645	1.75	0.160		19.3
Schooling below degree	0.320	1.05	0.32		30.4
Occupation (reference professionals)	0.004				
Unemployed	0.006	2.59	0.03		3.94
Semi-skilled worker	0.108	1.35	0.01		1.53
Skilled worker	0.017	2.4	0.00		0.57
Shop owner and farmer	0.004	1.4	0.03		0.34
Efficacy of dentist	0.007	2.8	0.114		0.715
Enabling resources (reference fair resources)	0.165				
No resources	0.436	1.74	0.08		2.38
Poor resources	0.436	1.45	0.43		7.08
Perceived need (reference poor)	0.001				
Good	0.002	1.20	0.03		0.45
Fair	0.001	2.05	0.91		0.54
DMFT	0.005	2.68	0.98		0.66
Past dental visits (reference no visit)	0.119	0.25	0.81		5.91
Prosthetic need (maxilla)	0.467	0.818	0.89		2.46

The study, conducted on a sample of 400 Gond tribes inhabiting confined villages in Chhattisgarh, provided comprehensive insights into the demographic factors and health beliefs influencing dental visits and perceived oral health outcomes within this population. Analysis revealed intriguing patterns in reasons for dental visits, with significant variations observed across different demographic groups. For instance, while the majority of participants sought dental care only when faced with a problem, significant disparities emerged based on factors such as age, gender, education, occupation, and socioeconomic status. Notably, individuals with higher education levels or belonging to certain occupational categories were more likely to seek dental care regularly or occasionally, indicating a potential link between education, occupation, and health-seeking behavior. Moreover, the study highlighted the significant impact of dental caries on dental visits, underscoring the importance of preventive dental care and early intervention strategies. Interestingly, while no significant associations were found between periodontal disease or loss of attachment and dental visits, prosthetic status and need exhibited varying associations, suggesting potential disparities in treatment utilization among individuals with different oral health conditions.

In addition to demographic factors, the study delved into the role of predisposing health beliefs in shaping dental visits and perceived oral health outcomes. The findings revealed significant associations between certain health beliefs, such as the efficacy of dentists and perceived barriers, and reasons for dental visits. These insights shed light on the complex interplay between individual perceptions, attitudes, and healthcare-seeking behaviors, emphasizing the importance of addressing both structural and attitudinal barriers to improve access to dental services.

Furthermore, the study examined the association between demographic factors and perceived oral health outcomes, revealing intriguing patterns in how age, education, occupation, and socioeconomic status influence individuals' perceptions of their oral health. Notably, enabling factors such as access to resources and perceived need for dental treatment emerged as significant predictors of perceived oral health outcomes, highlighting the importance of addressing systemic barriers and promoting awareness to enhance oral health outcomes among tribal communities.

Overall, the study's findings underscore the need for targeted interventions aimed at addressing disparities in dental care utilization and improving oral health outcomes among marginalized populations such as the Gond tribes in Chhattisgarh. By addressing both structural barriers and individual beliefs and perceptions, policymakers and healthcare providers can work towards ensuring equitable access to dental services and promoting better oral health outcomes for all members of the community.

## Discussion

The results presented in this study provide evidence in favor of Andersen's behavioral model of service utilization and health outcomes, particularly in perceived oral health. The study analyzes many social, attitudinal, and behavioral aspects that have significance in comprehending the circumstances surrounding the use of services for oral health within the particular tribal group. This study has incorporated the concept of predisposing health beliefs as a component within Andersen's model. It proposes that the objective to engage in a particular action arises from a combination of beliefs, including attitudes, norms, and perceived behavioral regulation. These characteristics serve as predictors of intentions, which in turn are associated with behavior. Individual attitudes and perceived norms are predicted by the demand for and availability of resources, which in turn determine the intention and subsequent actual usage of services [14]. On the other hand, an individual's health beliefs, attitudes, values, and knowledge regarding oral health and dental care may serve as a pathway through which social structural elements impact the availability of resources, the level of need, and the utilization of services [15]. Given the limited number of studies examining the usage of dental services across tribal populations, most comparisons are made with the general population as a whole.

The current study revealed that 86% of the participants had not sought dental care during the past year and none were seeking dental care within the past month. Concerning reasons to visit the dentist, the present study reported only when there is an oral health problem as the major (96.8%) reason to visit the dentist. The number of subjects visiting the dentist decreased significantly in the older age. This can be partly explained by the fact that as age increases people believe that dental problems are due to aging and ill health and other systemic conditions seem more important than oral health [16]. In the present study, the number of female participants was relatively greater as opposed to the number of males. This was congruent with the findings of an earlier study [17], while it was contrary to a previous study done by Nagarjuna P et al. [18].

Over 50% of the sample population had not received any sort of formal schooling, as demonstrated in the current study. This could be because the population is less aware of the importance of education and the failure of implementation of government educational programs in this area. Also, the burden of the financial crisis compels them to work to earn a livelihood rather than to spare time for education. The finding suggests that as the level of education increased, the number of subjects visiting the hospital also increased. Marriage significantly amplified the exponential growth of making decisions in prosthetic therapy (a 2.228-fold rise), with unmarried individuals serving as the baseline comparison group [19]. In contrast, the study done by McDonald did not show the impact of marital status on the utilization of dental care [20]. More past dental visits and more regular visits were seen with the higher occupation group. The reason for this may be that occupation and socioeconomic status are interrelated, which enables them to seek private dental treatments. Low perceived importance to oral health, unavailability of dentists nearby and lack of transport facilities, belief in home remedy, and misperception towards dental treatment were found as major barriers associated with low utilization of dental services [21,22].

Eliminating the barrier of expensive medical treatment can be achieved by the implementation of complementary health camps, which have demonstrated efficacy in disease screening and delivery of preventive treatment. It was seen that individuals with a fair and good perception of their oral health reported more regular or occasional dental visits in the past one year. Afonso-Souza et al. also found that people who rated their oral health as poor were substantially more inclined to disregard regular dental examinations in comparison to those who rated their oral health as good [23].

The study revealed that a majority of the individuals had a pocket depth of 4-5 mm (42.8%) and calculus (35.3%). The rise in periodontal disease among the participants could be attributed to inadequate oral hygiene practices, tobacco use, limited understanding of the state of oral health, and presumably indigenous brushing patterns. The results of the current study were consistent with a similar prior study [24]. In this study, CPI did not have a significant association with past dental visits (P>0.05) and reason to visit (P>0.05). The prevalence of dental caries was 80%, and the filled teeth were very low among Gond tribes, which indicates the dominance of untreated caries. Access to food, the type of diet they consume, low socioeconomic status, low level of education, lack of importance towards oral health, lacking enabling resources, and barriers to access oral healthcare are some factors that result in high caries prevalence [25]. These findings indicate high treatment needs among the population and less utilization of oral health services. A study undertaken by Doughan et al. among individuals from Lebanon found similar results to the current study, indicating that the participants had a higher requirement for prostheses due to their dearth of understanding regarding tooth replacement [26].

The present study disclosed that the perceived oral health outcome was significantly associated with past dental visits, whereas there was an insignificant association found between perceived oral health outcome and reason to visit. Among the predisposing factors, only age and occupation showed a significant association with perceived oral health outcomes. Middle-aged adults had 2.8 times the odds of having good perceived oral health outcomes as compared to the elderly. This finding is similar to the finding of previous studies, which showed that as the age advances the perceived oral health outcome becomes poor [27]. The participants who perceived higher efficacy of dentists had 2.8 times the odds of having good perceived oral health outcomes. This was congruent with the finding of a study conducted by Broadbent et al., which explains that people with a favorable belief in the efficacy of dentists have much better clinical conditions and self-rated oral health [28]. The findings showed that individuals with fair perceived oral health had 2.1 times the odds of having a good perceived oral health outcome and individuals with good perceived oral health had 1.2 times the odds of having a good perceived oral health outcome as compared to poor perceived oral health. This finding was in line with the previous study conducted by Massod et al., where people with poor self-reported health had 2.3 times higher odds of poor perceived oral health outcomes than the participants with good self-reported health [29]. The results revealed that the individuals with more untreated dental caries had 2.6 times the odds of having a good perceived oral health outcome. The study conducted by Papaioannou et al. also showed similar findings [30].

The study has some limitations such as the data gathered in the current cross-sectional study were analyzed according to the causal sequencing proposed in Andersen's model. However, it is important to note that this grouping does not necessarily indicate a causal relationship. In order to delve deeper into intricate processes, subsequent investigations must adopt an approach that is longitudinal. The perceived need for therapy is known to impact the decision to seek treatment (utilization of healthcare services) and the other way around. Incorporating the examination of such mutual interactions is necessary for future investigations. Moreover, a limitation of OHIP-14, which is employed to evaluate perceived oral health outcomes, concerns its potential failure to sufficiently account for variations in anticipations across different geographical areas. Consequently, this could lead to inaccurate assessment of perceived oral health outcomes either through downplaying or exaggerating its importance depending on the specific group being studied. Ultimately, the conclusions on service utilization are likely specific to the belief system, culture, and organizational framework of services accessible to the Gond tribes of Chhattisgarh. The cross-validation of the findings should encompass samples from more tribal groupings and diverse states. Also, the challenges of reducing long-standing oral health disparities, expanding access to oral health services at affordable prices, and keeping up with the quality of the treatment are to be dealt with judiciously by government officials and health policymakers to strengthen the Indian oral health system.

## Conclusions

In summary, our study highlights insufficient utilization of oral healthcare services among participants, indicating a lack of priority given to oral health. Positive beliefs and behaviors, such as recognizing the importance of dentists and understanding the seriousness of oral diseases, are associated with higher service utilization. Factors like proximity to dental services, affordable treatment costs, and reduced dental fear contribute to more equitable utilization. Individuals who acknowledge their dental needs and perceive dentists as effective are more likely to seek care. However, many participants underestimate their oral health needs. To improve service utilization and oral health outcomes, addressing barriers to access and increasing awareness are essential steps.
